# Predicting prognosis and clinical features of the tumor microenvironment based on ferroptosis score in patients with breast cancer

**DOI:** 10.1038/s41598-022-14964-7

**Published:** 2022-06-23

**Authors:** Lianli Yin, Yinghua Tang

**Affiliations:** 1 Department of Clinical Laboratory, The People’s Hospital of Guangxi Zhuang Autonomous Region, Guangxi Academy of Medical Sciences, No. 6 Taoyuan Road, Nanning, Guangxi 530021 China; 2 Department of Clinical Laboratory, Guangxi Hospital of Traditional Chinese Medicine, The First Affiliated Hospital of Guangxi University of Chinese Medicine, Nanning, Guangxi 530023 China

**Keywords:** Breast cancer, Breast cancer, Cancer microenvironment

## Abstract

Ferroptosis genes have recently been reported to be involved in regulating the development of cancer, but their potential role in breast cancer (BRCA) is not fully understood. The purpose of this study is to systematically study the mechanism of ferroptosis in BRCA and its relationship with this cancer’s prognosis, cell infiltration, gene mutation, and other clinical features. In this study, The Cancer Genome Atlas breast cancer (TCGA-BRCA) database (UCSC Xena) was used to mine the ferroptosis genes related to BRCA patients, and the genes with prognostic value were screened by Cox regression analysis, which were then used to construct a prognostic model for scoring prognostic molecular risk. The relationships between ferroptosis score and prognosis, molecular typing, and clinical characteristics of BRCA were also analyzed. A total of 176 ferroptosis genes related to BRCA were retrieved from the database, 22 of which were found to be significantly related to BRCA prognosis after screening by single-factor Cox regression analysis (*p* < 0.01). Unsupervised clustering of samples was performed using factoextra, and two subgroups (ferroptosis cluster A and ferroptosis cluster B) with significant differences in prognosis were identified. Subsequently, single-factor Cox regression analysis and random forest dimensionality reduction were used to screen characteristic genes to construct a ferroptosis score model, which included a high ferroptosis score group and a low ferroptosis score group. The results showed that there were significant differences in ferroptosis scores between the ferroptosis cluster A and B groups. The prognosis of patients with low ferroptosis scores was poor, and the overall survival (OS) rate of patients with high ferroptosis scores was significantly higher, indicating that the prognosis of the sample can be well characterized based on calculated ferroptosis scores. Ferroptosis scores differed significantly according to patient age, TP53 and PIK3CA gene mutations, different PAM50 molecular types, and clinical stages. Ferroptosis activation plays a non-negligible role in tumor occurrence and development. Evaluating the ferroptosis score within BRCA will help advance our understanding of the infiltrating properties of cells in the tumor microenvironment and may guide more effective immunotherapy strategies.

## Introduction

Breast cancer (BRCA) has become one of the most common malignant tumors in women globally, and it is the type of malignant tumor with the highest mortality in women^1^. Recently, its morbidity and mortality have increased year by year, and it has become the second most frequently diagnosed disease in the world^2^. A trend has been observed that the disease is gradually affecting younger patients, which is a serious threat to women’s health^3,4^. The etiology and mechanism of BRCA are not fully understood, but gender, race, age, genetics, early menstruation, and late menopause are factors that lead to an increased risk of BRCA, and clinical features such as cell infiltration and genetic mutations are the main factors leading to the spread and metastasis of BRCA. Therefore, early detection and prognostic evaluation are the best methods to prevent and treat the spread of BRCA.

With the deepening of clinical research on the diagnosis, prevention, treatment, and prognosis of BRCA have received extensive attention. More and more molecular biological indicators are being used to assess the prognosis of patients, to guide their treatment, and to determine the effects of treatment. Ferroptosis is a recently identified mode of programmed cell death that differs from necrosis and apoptosis. Ferroptosis refers to cell death caused by the accumulation of lipid reactive oxygen species due to the inactivation of glutathione peroxidase 4 (GPx4)^5,6^. Studies have found that in liver cancer, pancreatic cancer, prostate cancer, and BRCA, ferroptosis can inhibit the proliferation of tumor cells and that the activation of ferroptosis can accelerate the progression of neurodegenerative diseases such as Alzheimer’s disease^7^. It can be seen that ferroptosis plays an important role in tumor treatment, and the induction of ferroptosis in cancer cells has gradually become a tumor treatment strategy^8^.

At present, basic research on the induction of ferroptosis in tumor cells for the treatment of BRCA has gradually shown good results, and it is expected to provide new treatment options for BRCA patients^9^. Screening and preventing illness in the vulnerable population is more conducive to the early diagnosis and treatment of BRCA, and it is of great significance for controlling the condition of BRCA^10^. This can be done by analyzing the clinicopathological characteristics of BRCA patients and mastering their epidemiological characteristics. In this study, we quantified the ferroptosis activation pattern of a single BRCA sample. Based on the ferroptosis-related genes, a prognostic model of ferroptosis score risk assessment was established for BRCA patients to reveal the clinical prognostic value of ferroptosis scores and to analyze the relationships between ferroptosis score and prognosis, molecular typing, and clinical characteristics of BRCA. This model is intended to provide a new perspective on the analysis of BRCA prognosis.

## Materials and methods

### Source and preprocessing of breast cancer dataset

The gene mutation, copy number variation, expression profile (RNASeq), and clinical data of The Cancer Genome Atlas breast cancer (TCGA-BRCA) were obtained from the University of California Santa Cruz (UCSC) Xena database (http://xena.ucsc.edu/). In the Gene Expression Omnibus (GEO) database (https://www.ncbi.nlm.nih.gov/geo/) number GSE20685, the expression profile of the BRCA population and clinical data were selected, and the GEO. The expression profile and clinical data of French BRCA patients were obtained from the International Cancer Genome Consortium (ICGC) database (https://dcc.icgc.org/releases/current/Projects/BRCA-FR), named ICGC-BRCA-FR. The samples from male patients, from patients with no clinical survival information, and from patients with OS time of less than one month from the three data sources were removed. Details about the final sample are shown in Table 1. Because the data come from TCGA, GEO, and ICGC, no additional permission from the ethics committee is required, and all methods were performed in accordance with the relevant guidelines and regulations.

**Table 1:** Sample information.

	Dataset	TCGA	GSE39582	GSE41258
Stage	I	153	69	20
	II	510	147	53
	III	190	103	21
	IV	20	8	0
	N/A	170	0	4
OS status	Deceased	153	83	4
	Living	890	244	94
Age	> 65	296	22	20
	≤ 65	747	305	78
PAM50	Basal	133		
	Her2	61		
	LumA	402		
	LumB	181		
	Normal	23		
TP53	Mut	327		
	WT	622		
	N/A	94		
PIK3CA	Mut	332		
	WT	617		
	N/A	94		

### Data preprocessing

To maintain data consistency, a subset of each expression profile according to the common gene was first taken, and then the data were merged. Subsequently, the batch effect was removed using the combat method of the SVA (version 3.42.0) R software package^11^. The combined dataset contained 1,468 samples and 15,343 gene expression profiles (Supplementary Table 1), including 1,043 samples from TCGA-BRCA, 327 samples from GSE20685, and 98 samples from ICGC-BRCA-FR.

#### Unsupervised clustering using ferroptosis genes

A total of 176 ferroptosis-related genes (Table 2, Supplementary Table 2) were collected through literature and database searches^12–15^. Cox regression analysis was then used to screen the ferroptosis genes significantly related to prognosis for subsequent analysis (*p* < 0.01), ultimately obtaining 22 ferroptosis genes (Table 3). The hcut method of Factoextra (version 1.0.7) was used to perform unsupervised clustering of samples. The expression data of these genes were extracted and used to identify the pattern of ferroptosis gene expression. Based on the expression of these 22 ferroptosis-related genes, different ferroptosis activation modes were identified. The R-package Consensus Cluster Plus (version 1.50.0) was then used to perform an unsupervised cluster analysis of ferroptosis gene expression^16^.

**Table 2:** Ferroptosis related genes.

No	Ferroptosis-related genes	Name
1	ACSL4	acyl-CoA synthetase long-chain family member 4
2	AKR1C1	aldo–keto reductase family 1 member C1
3	AKR1C2	aldo–keto reductase family 1 member C2
4	AKR1C3	aldo–keto reductase family 1 member C3
5	ALOX15	arachidonate 15-lipoxygenase
6	ALOX5	arachidonate 5-lipoxygenase
7	ALOX12	arachidonate 12-lipoxygenase
…	…	…
176	CRYAB	crystallin alpha B

Due to space reasons, only some genes are listed. For detailed information, see Table 2.xlsx in the supplementary.

**Table 3:** Results of ferroptosis gene Cox regression analysis.

No	Ferroptosis gene	Cox. *p* value
1	PANX1	8.20E–06
2	MTDH	4.17E–05
3	FLT3	6.03E–05
4	IFNG	0.000132
5	CISD1	0.000157
6	G6PD	0.000219
7	ATG5	0.000481
8	MYB	0.000516
9	CA9	0.00158
10	SQLE	0.00161
11	PHKG2	0.00174
12	ACSF2	0.00263
13	ALOX15	0.00265
14	TP63	0.0027
15	LAMP2	0.00297
16	CS	0.0032
17	ALOX5	0.00415
18	HIF1A	0.00514
19	VDAC1	0.00523
20	MAP1LC3A	0.00579
21	TGFBR1	0.00607
22	ALOX15B	0.00949

### Gene set variation analysis (GSVA) and single sample gene set enrichment analysis (ssGSEA)

To further study the different ferroptosis activation patterns in biological processes, the c2.cp.kegg.v7.1 gene set was downloaded from the MsigDB database (https://www.gsea-msigdb.org/gsea/msigdb/index.jsp) for enrichment analysis of gene set variation analysis (GSVA). GSVA is a non-parametric, unsupervised method mainly used to estimate changes in pathways and biological process activity in samples. R-package GSVA was used for enrichment analysis of the Kyoto Encyclopedia of Genes and Genomes (KEGG) pathway^17–19^. Then, the single sample gene set enrichment analysis (ssGSEA) method was used to analyze and obtain the infiltration ratio of 23 immune cells^20,21^. The Wilcoxon test was used to compare the differences between ferroptosis cluster samples to evaluate the proportion and difference of these 23 immune cell types in the clusters. Then, the prognostic analysis of cells that showed differences (*p* < 0.05) was carried out by univariate Cox regression analysis, and the prognostic differences were compared at the same time.

### Identification of differentially expressed genes among different ferroptosis clusters

Based on ferroptosis cluster, BRCA samples were divided into ferroptosis cluster A and ferroptosis cluster B. The R-package limma (version 3.41.18) was used to identify the differentially expressed genes among different ferroptosis cluster^22^. The significance standard for identifying differential genes was p.adj < 0.01&|log2(FC)|> log2(1.5), where p.adj represents the *p* value after Bonferroni-Holm correction, and log2(FC) represents the value of the fold change of the mean between groups transformed into the base logarithm by 2.

### Ferroptosis score calculation

First, univariate Cox regression analysis was conducted for the differential genes obtained in the previous analysis to screen for genes significantly related to prognosis (false discovery rate adjusted *p* < 0.01). Redundant genes were then removed using the random forest method. Finally, principal component analysis of the expression profile data of the remaining genes was conducted with reference to the scoring standard of GGI^23^, and ferroptosis scores were calculated using the following formula:

$$ {\text{ferroptosis score }} = \sum {(PC1_{i} + PC2_{i} )} $$

where i represents the expression level of genes related to the ferroptosis phenotype.

Based on the obtained ferroptosis score results, the samples were divided into two equal groups: high ferroptosis score and low ferroptosis score. The correlation between the two types of samples and prognosis was further analyzed.

### Correlation between ferroptosis score and other biological processes

Mariathasan et al.^24^ screened for a set of genes related to certain biological processes, including EMT1, EMT2, and EMT3 pathways, immune checkpoints, antigen processing and presentation, and other epithelial-mesenchymal transition markers. Our study conducted a Pearson correlation analysis of ferroptosis scores and these biological processes, which further revealed the correlation between ferroptosis score and related biological pathways.

### Statistical analysis

The Fisher’s exact test was used to compare the proportion differences between the groups. The Kaplan–Meier curve and log-rank test were used to compare the OS of different risk groups. Univariate and multivariate Cox regression analyses were used to determine independent predictors of OS. In the figures of all statistical tests, ns means *p* > 0.05, * means *p* < 0.05, ** means *p* < 0.01, and *** means *p* < 0.001. All reported *p*-values were obtained by two-way tests, and *p*-values < 0.05 were considered statistically significant. All statistical work was done in R (version 4.1.0) environment.

## Results

### Unsupervised clustering of ferroptosis genes in BRCA samples

**Figure 1:**
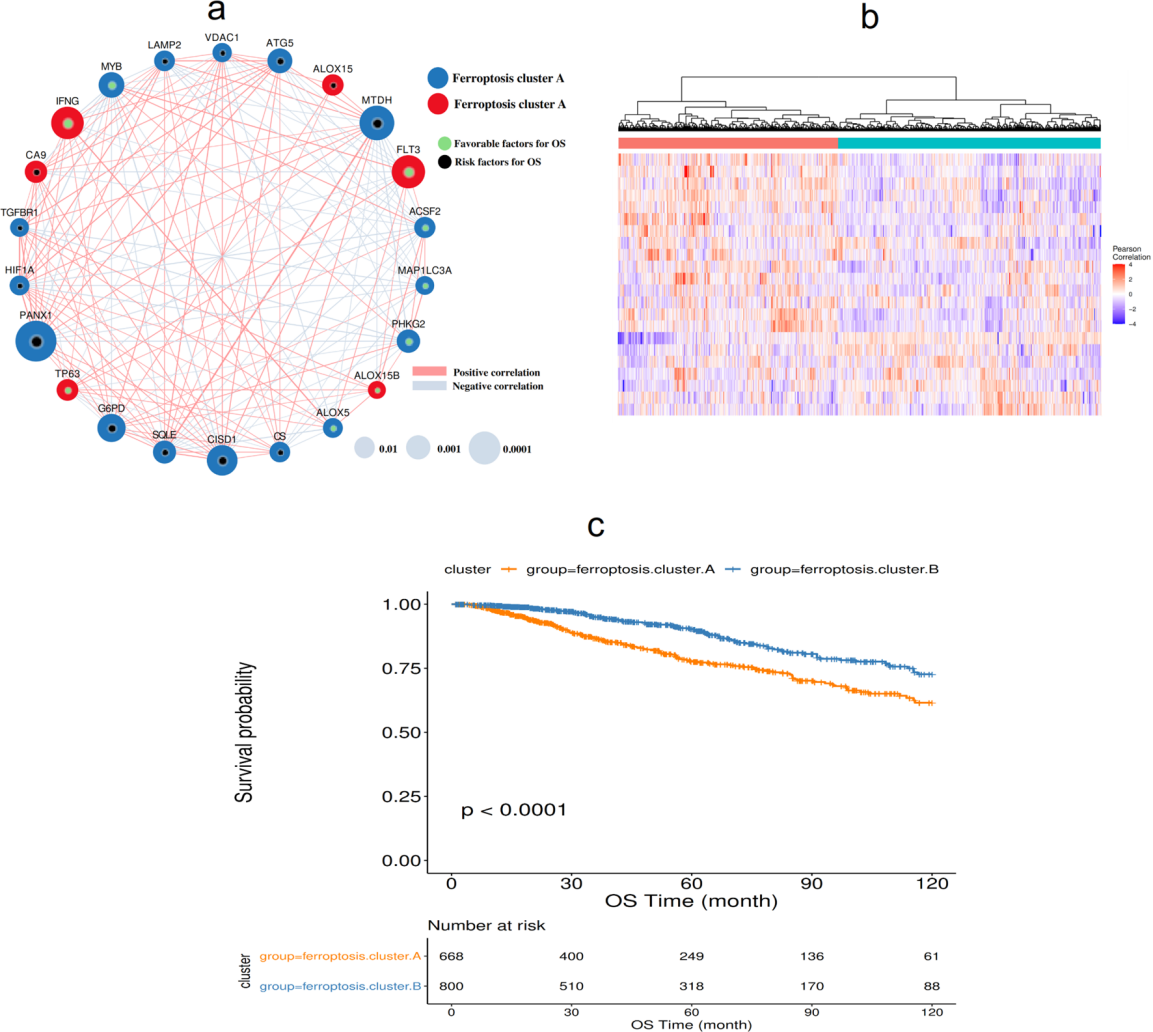
Expression of ferroptosis genes in BRCA. (**a**) The interaction between ferroptosis genes. The size of the circle represents the impact of each gene on survival prediction (the larger the circle, the more relevant the gene to survival). The green dots in the circle represent protective prognostic factors, and the black dots in the circle represent prognostic risk factors; (**b**) Unsupervised clustering of ferroptosis genes; (**c**) Kaplan–Meier curve showing a significant difference in survival between the two ferroptosis clusters.

**Figure 2:**
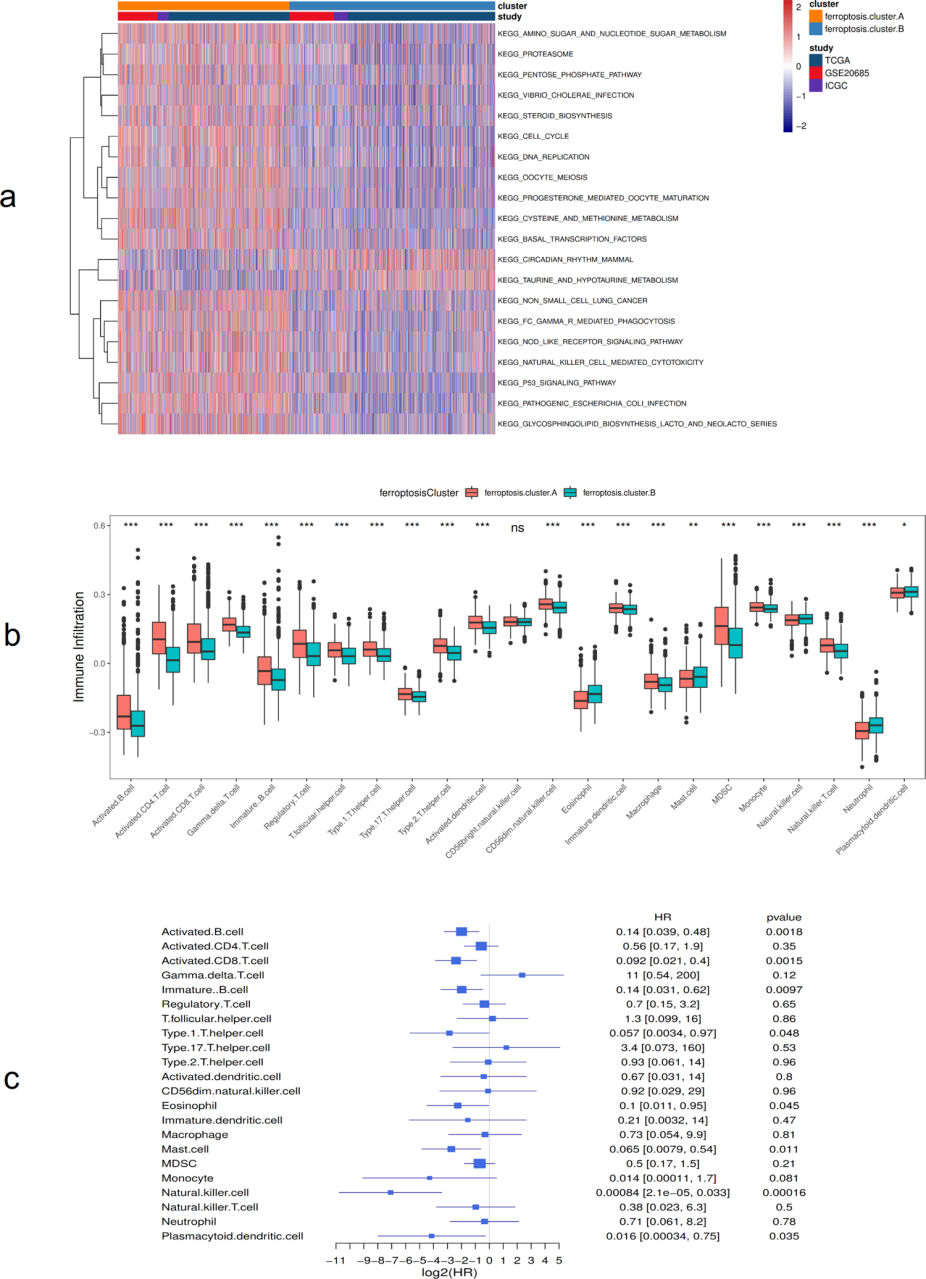
Biological behavior among ferroptosis expression patterns. (**a**) Gene Set Variation Analysis (GSVA) enrichment analysis results (different ferroptosis clusters show the activation status of biological pathways. Red indicates activation and blue indicates inhibition). *p < 0:05, **p < 0:01, and ***p < 0:001; (**b**) The distribution of immune infiltration of 23 kinds of immune cells in different ferroptosis clusters; (**c**) Prognosis analysis of differential cells.

First, Cox single factor regression analysis was used to screen for ferroptosis genes that were significantly related to the prognosis of BRCA, with a screening threshold of *p* < 0.01, and 22 genes were obtained for subsequent analysis (Table 3). The ferroptosis network describes the interactions between ferroptosis genes (Fig. 1a). The expression profiles of 22 ferroptosis genes were extracted, and then a consistent cluster analysis of these expression profiles was carried out. Finally, two subgroups of these ferroptosis genes were identified: ferroptosis cluster A and ferroptosis cluster B (Fig. 1b). The two subgroups showed significant differences in prognosis (*p* < 0.0001, Fig. 1c). By analyzing the differences in the expression profiles of cluster A and cluster B, 516 differential genes were obtained (Supplementary Table 3). To explore the biological behavior of these different ferroptosis expression patterns, the KEGG pathway was enriched and analyzed using R-package GSVA. The results showed that ferroptosis cluster A was significantly enriched in such biological pathways as DNA replication and cell cycle. Ferroptosis cluster B was significantly enriched in such biological pathways as taurine metabolism (Fig. 2a).

Then, the infiltration ratios of 23 kinds of immune cells were obtained by ssGSEA. The results are shown in Fig. 2b. The immune cells were distributed in different proportions in different ferroptosis clusters, except for cd56bright.natural.killer.cell. Subsequently, single-factor Cox analysis was performed on the immune cells that showed different cell ratios between the two ferroptosis cluster groups. The results showed that the cell prognoses of Natural.killer.cell, Activated.CD8.T.cell, Activated.B.cell, and Immature.B.cell are quite different (Fig. 2c, Supplementary Table 5, 6).

**Figure 3:**
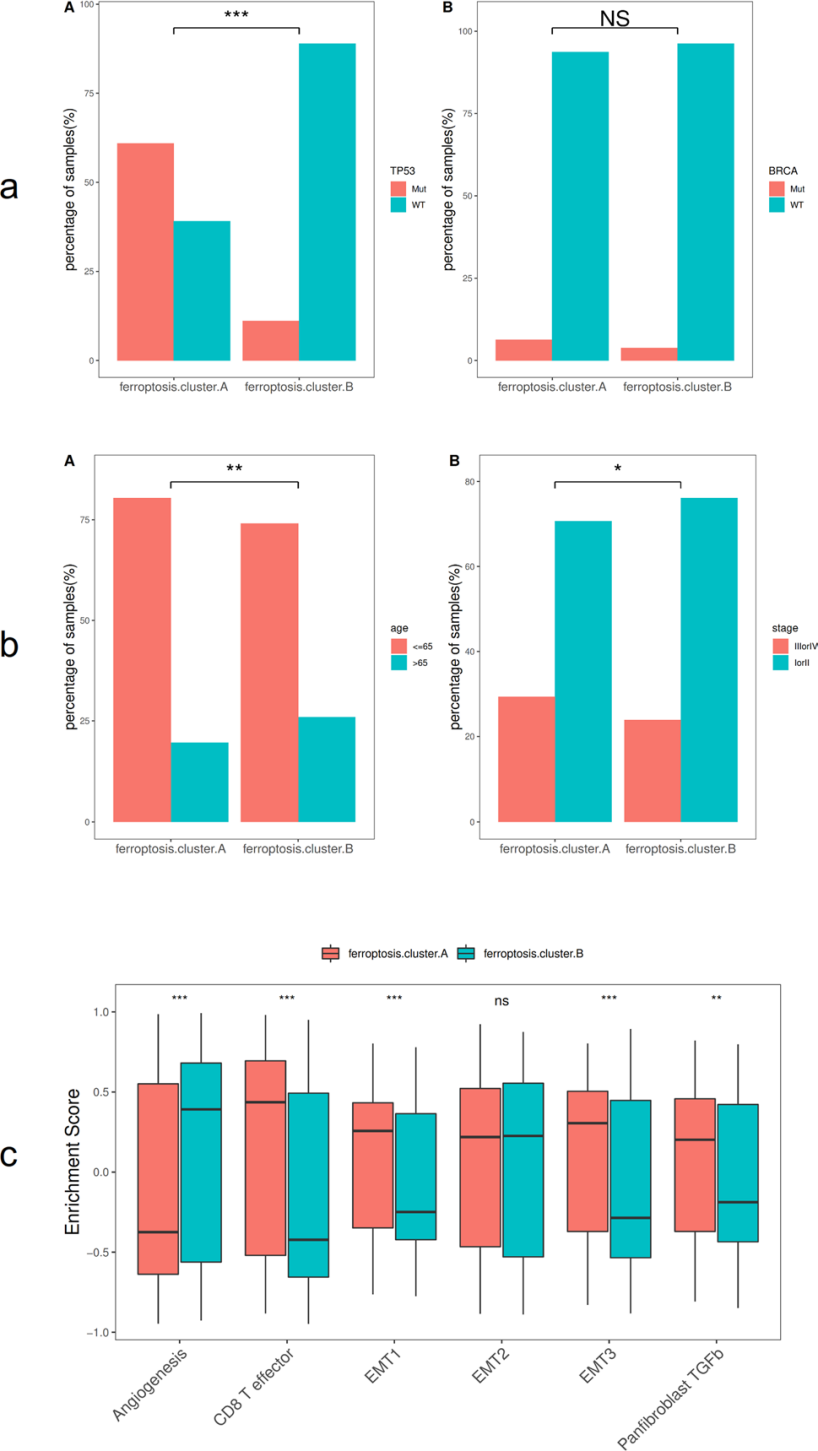
Association analysis between ferroptosis clusters and clinical characteristics. (**a**) Comparative analysis of ferroptosis clusters in the TCGA-BRCA data set (A: TP53 mutation, B: BRCA); (**b**) Comparative analysis of ferroptosis clusters in the TCGA-BRCA dataset (A: age, D: cancer stage); (**c**) Differences in enrichment scores of different ferroptosis clusters, *p < 0:05, **p < 0:01, and ***p < 0:001.

**Figure 4:**
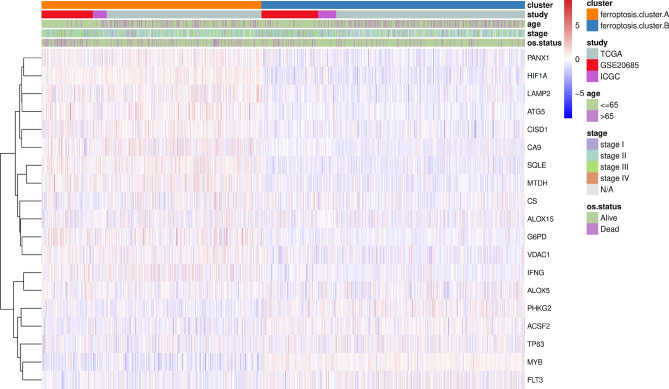
The expression of ferroptosis genes in ferroptosis clusters.

In the TCGA dataset, the chi-square test was used to statistically measure the mutation frequency of common tumor driver genes in the ferroptosis cluster A and ferroptosis cluster B subgroups. A significant difference was found in the frequency of the TP53 gene, but there was no difference in BRCA (BRCA1/BRCA2) (Fig. 3a). The ferroptosis cluster subgroups showed significant differences in terms of clinical stage and patient age, patients older than 65 years and with less advanced tumor stages were more concentrated in the high ferroptosis score group (Fig. 3b). Subsequently, this study used the gene set constructed by Mariathasan et al. to perform GSVA analysis. The results showed that the ferroptosis cluster groups had significant differences in cytokine enrichment scores, in addition to EMTA 2 (Fig. 3c). The distribution of ferroptosis genes in ferroptosis cluster A and ferroptosis cluster B is shown in Fig. 4.

### Differential genes of ferroptosis clusters

**Figure 5:**
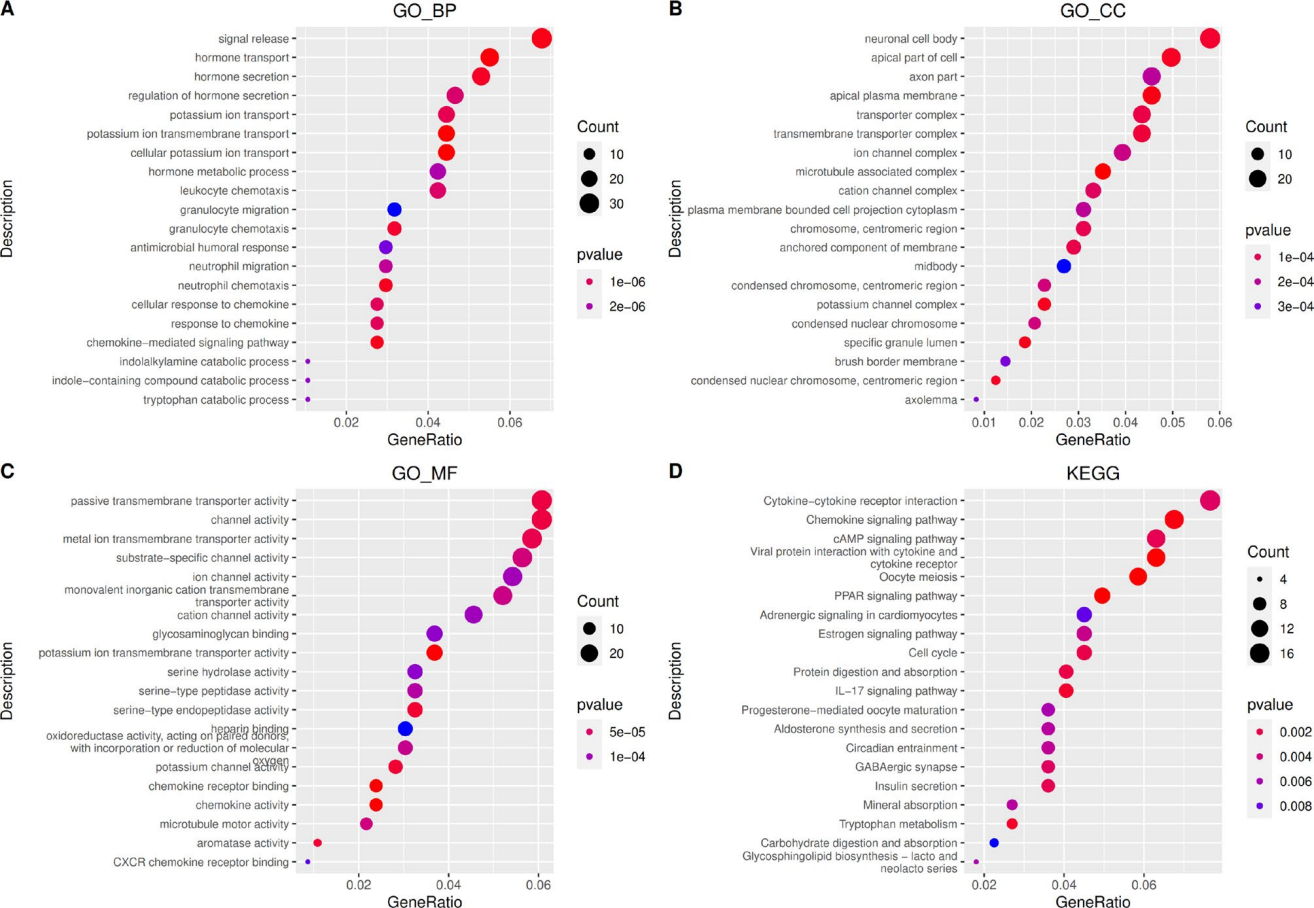
Differential gene function and pathway enrichment analysis results (top 20 with significant differences). (**A**) Biological process (BP); (**B**) Cellular component (CC); (**C**) Molecular function (MF); (**D**) Kyoto Encyclopedia of Genes and Genomes(KEGG) pathway enrichment analysis.

To further study the potential biological behavior of each ferroptosis cluster, 516 differential genes related to ferroptosis phenotypes were determined using limma software. Based on the R package clusterProfiler (version 3.13.0), GO (Gene ontology) function and KEGG (Kyoto Encyclopedia of Genes and Genomes) pathway enrichment analysis were performed^25–27^. It was found that differential genes are mainly enriched in biological processes such as hormone transport and hormone secretion, as well as in pathways such as cytokine–cytokine receptor interaction (Fig. 5).

**Figure 6:**
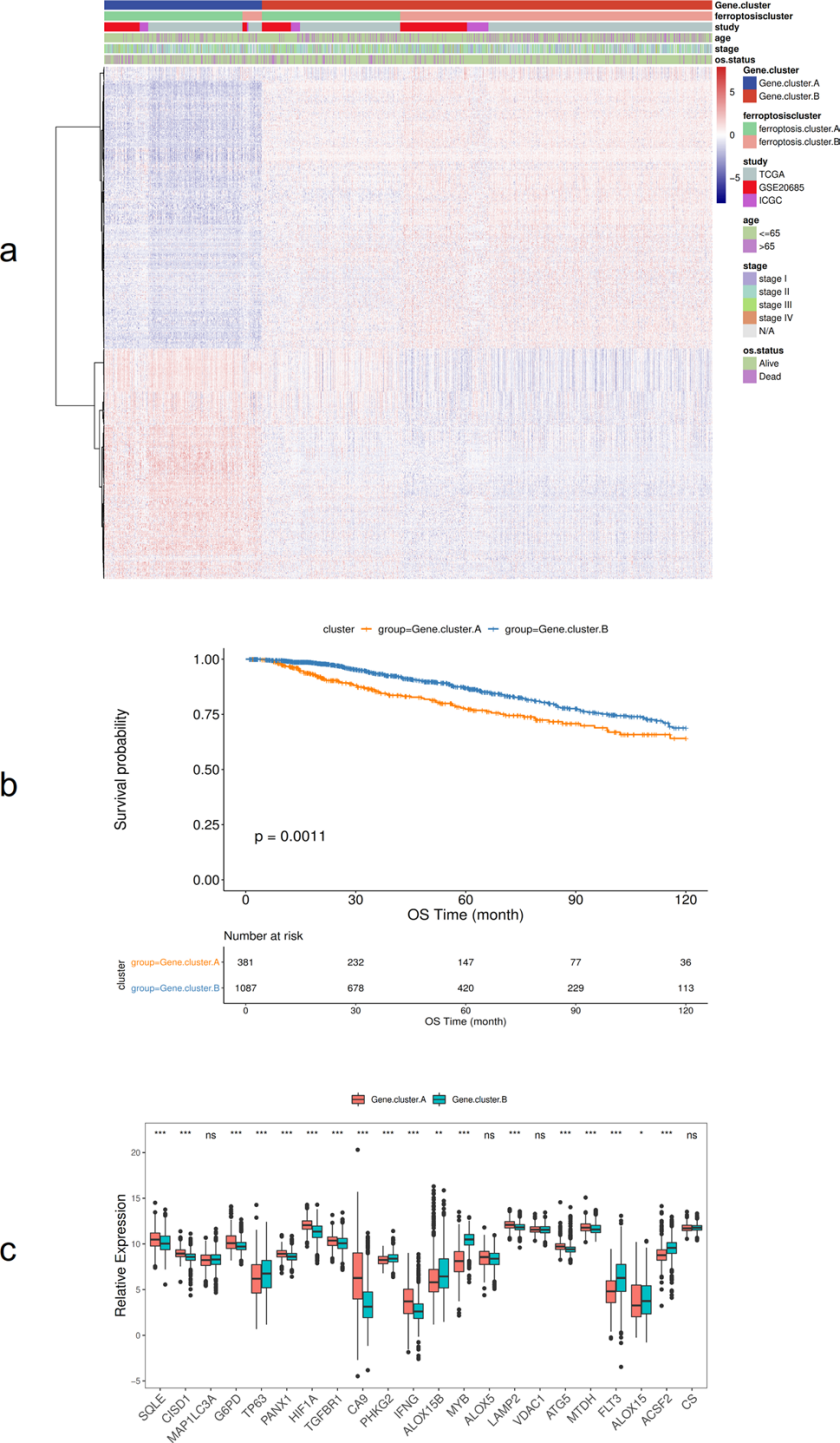
Association of ferroptosis with other datasets and prognosis. (**a**) Unsupervised clustering of genes related to ferroptosis phenotypes in BRCA samples; (**b**) Kaplan–Meier curve showing that ferroptosis gene clusters have a significant relationship with overall survival rate; (**c**) The expression of 22 ferroptosis genes in two gene clusters, *p < 0:05, **p < 0:01, and ***p < 0:001.

The samples were divided into different genomic subtypes by unsupervised cluster analysis according to the different genes in the ferroptosis clusters. Consistent with the cluster grouping of ferroptosis gene expression patterns described in 3.1, the unsupervised clustering algorithm also revealed two different ferroptosis genome phenotypes, called ferroptosis gene cluster A and ferroptosis gene cluster B (Fig. 6a). The expression of most ferroptosis genes is significantly different between ferroptosis gene cluster groups (Fig. 6b). It was observed in the model that the type of tumors associated with ferroptosis gene cluster A have poor prognosis (Fig. 6c).

### Ferroptosis score analysis

**Figure 7:**
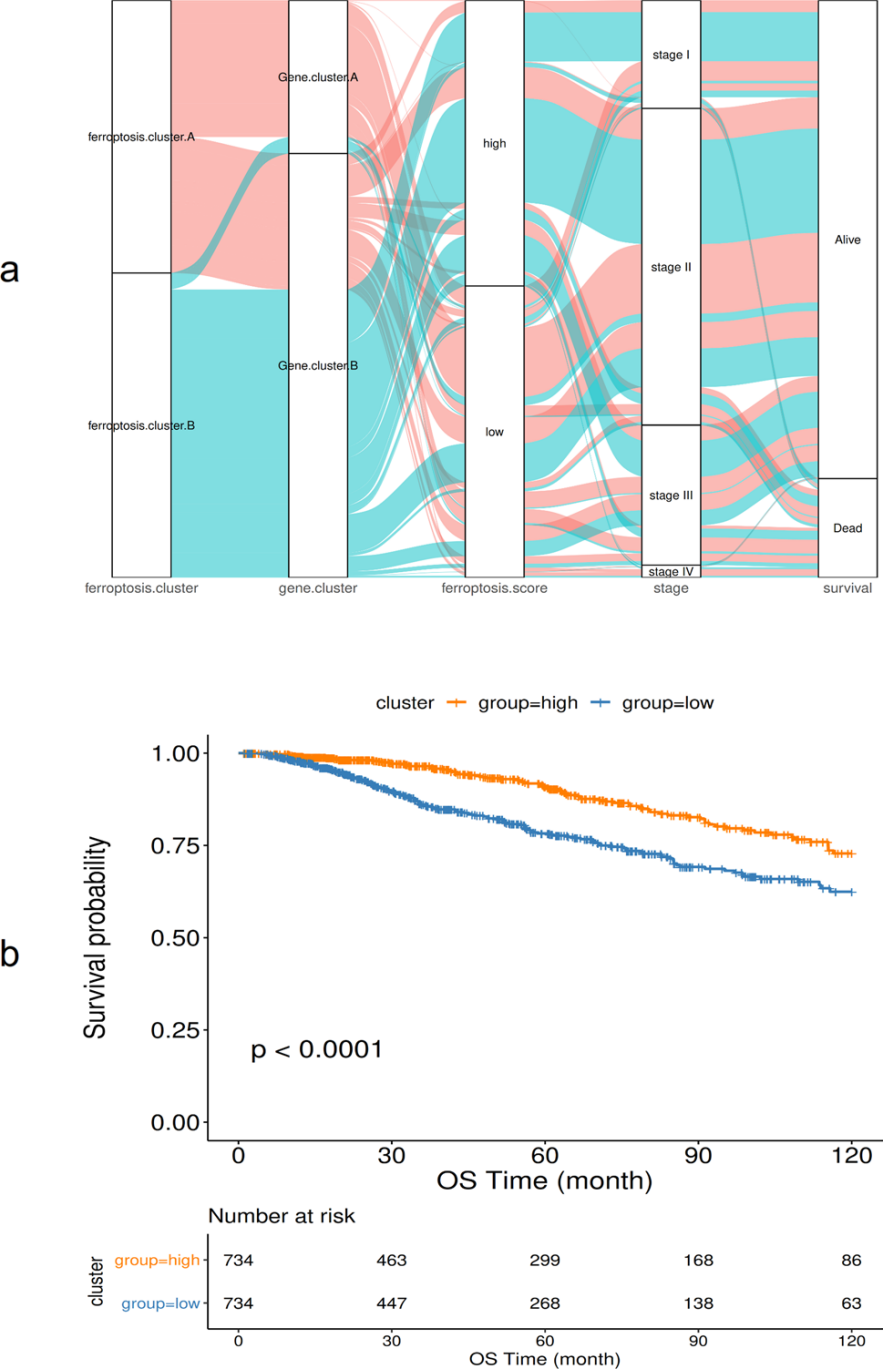
The relationship between ferroptosis score and patient prognosis. (**a**) Alluvial diagram showing changes in ferroptosis clusters, gene clusters, disease stages, and ferroptosis score; (**b**) Kaplan–Meier curve showing a significant relationship between the high and low ferroptosis score groups and overall survival rate.

Of the 516 differential genes obtained in the previous step, redundant genes were removed according to the correlation between the genes. Then, a single-factor Cox regression model was used to determine the relationship between these genes and the prognosis of the sample, and the insignificant genes were filtered out (FDR adjusted *p* ≥ 0.01). The random forest algorithm was used to identify the characteristic genes most relevant to ferroptosis phenotype. Principal component analysis was performed on the expression profiles of the remaining 9 ferroptosis phenotype–related genes (BCL2, SUSD3, SERPINA3, AGBL2, SEC14L2, ELOVL2, FGD3, CASC1, TPRG1 (Supplementary Table 4)), and then the ferroptosis score calculation formula was used to calculate the ferroptosis scores of all samples. Finally, the samples were sorted according to ferroptosis score and divided into two groups: high ferroptosis score and low ferroptosis score. As shown in Fig. 7a, ferroptosis cluster A corresponds mainly to low ferroptosis score, and it comprises mainly more advanced tumor stages. Similarly, ferroptosis cluster B corresponds mainly to high ferroptosis score, and it comprises mainly less advanced tumor stages. Kaplan–Meier curve analysis showed that the OS rate of the low ferroptosis score group was lower than that of the high ferroptosis score group (*p* < 0.0001), indicating that low ferroptosis score indicates poor prognosis (Fig. 7b).

**Figure 8:**
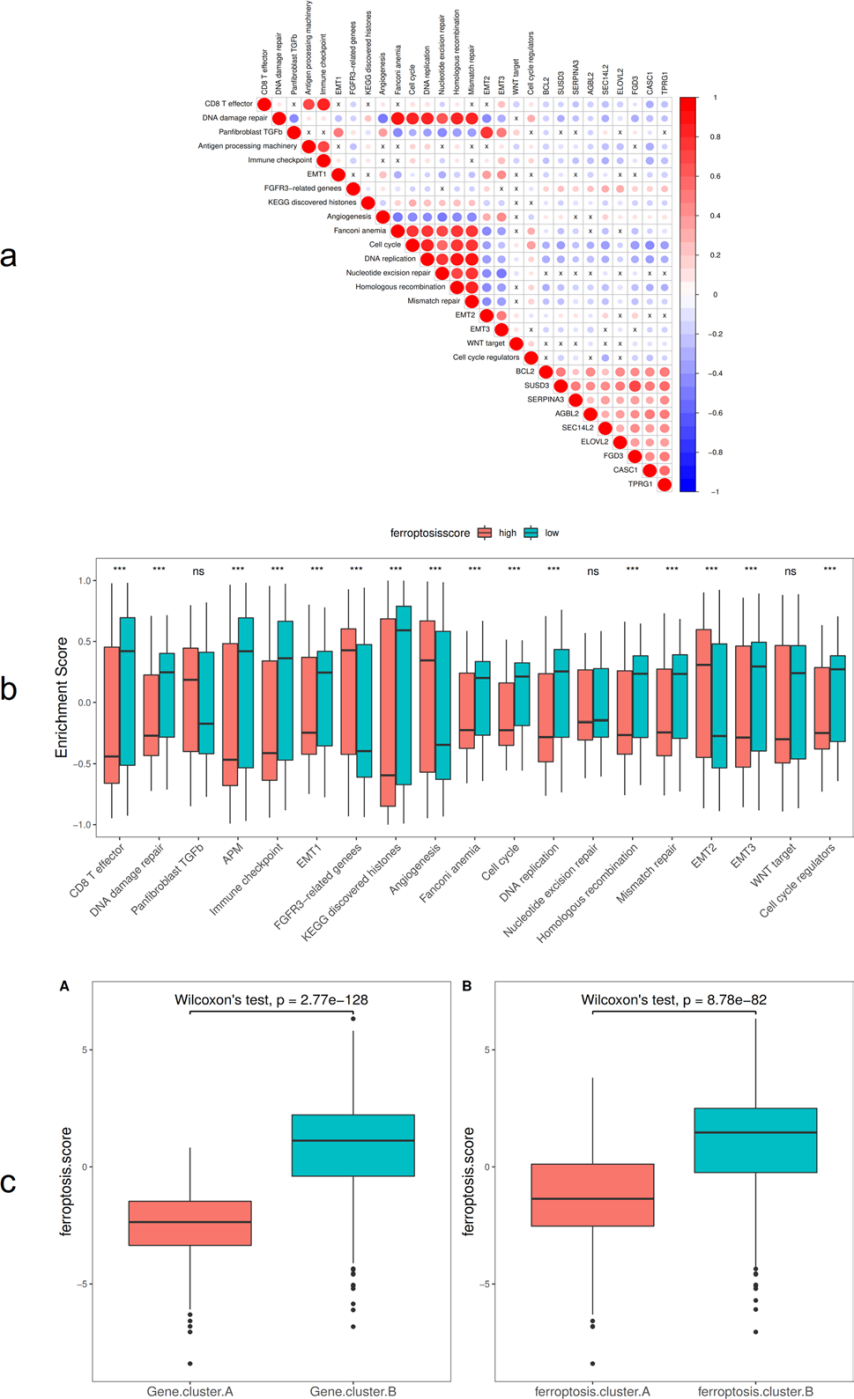
Ferroptosis score and pathway correlation analysis. (**a**) Correlation between ferroptosis feature gene, ferroptosis score, and known biological pathways; (**b**) The distribution of enrichment scores of known gene characteristics in the BRCA dataset in high and low ferroptosis score groups, *p < 0:05, **p < 0:01, and ***p < 0:001; (**c**) The distribution of ferroptosis scores in ferroptosis gene cluster A and ferroptosis cluster B.

Further analysis of the correlation between ferroptosis score and known gene features constructed by Mariathasan et al. showed that ferroptosis score was significantly positively correlated with biological functions such as DNA replication, cell cycle, and DNA mismatch repair (Fig. 8a). Moreover, the enrichment scores of known gene features in most BRCA data sets showed statistically significant differences between low and high ferroptosis score group samples (*p* < 0.05 (Fig. 8b)). The Wilcoxon test showed that ferroptosis clusters and ferroptosis gene clusters also had significant differences in ferroptosis scores (*p* < 0.05, (Fig. 8c)).

**Figure 9:**
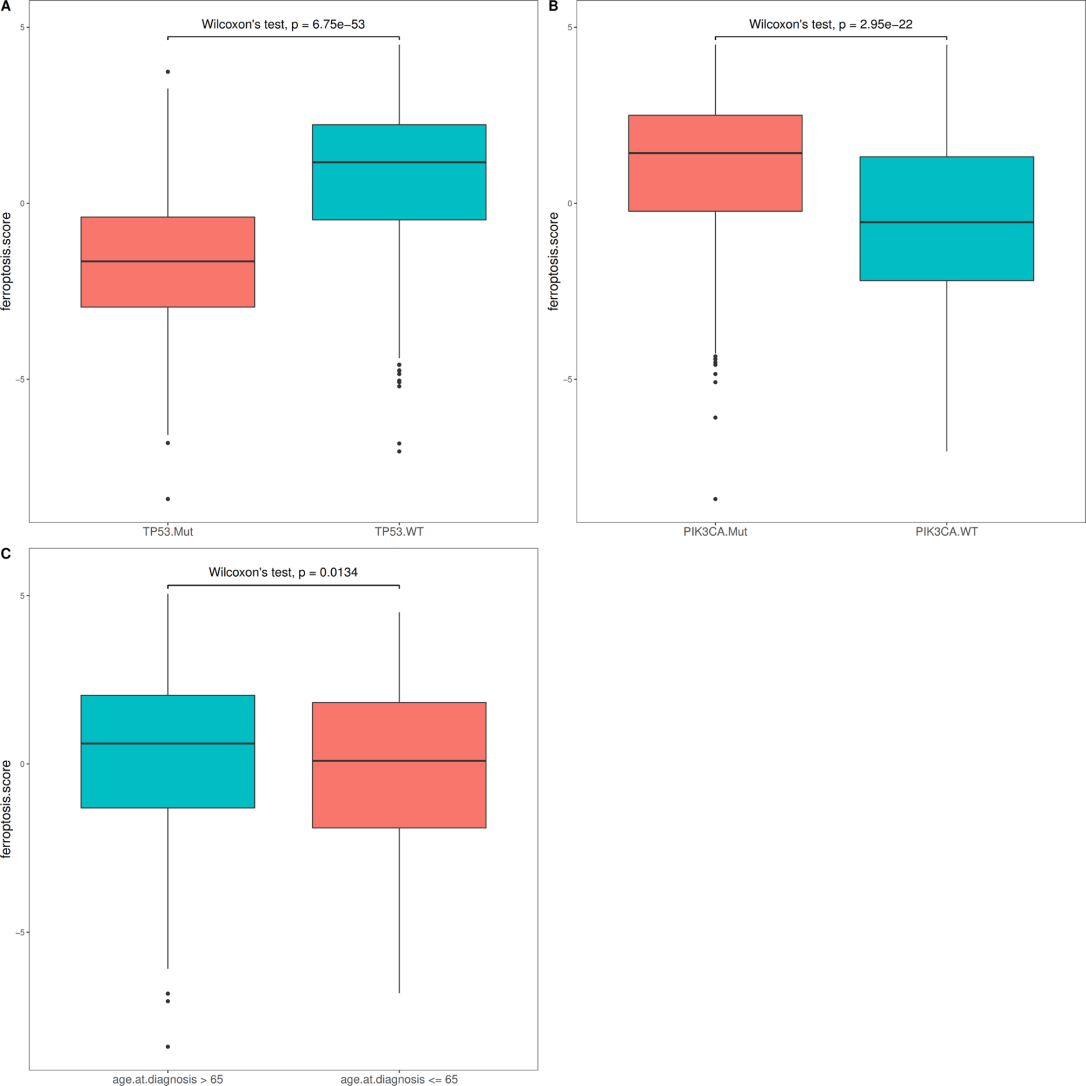
Comparative analysis of ferroptosis scores in different molecular subtypes and clinical characteristics (**A**: TP53 mutation; **B**: PIK3CA mutation, **C**: age).

**Figure 10:**
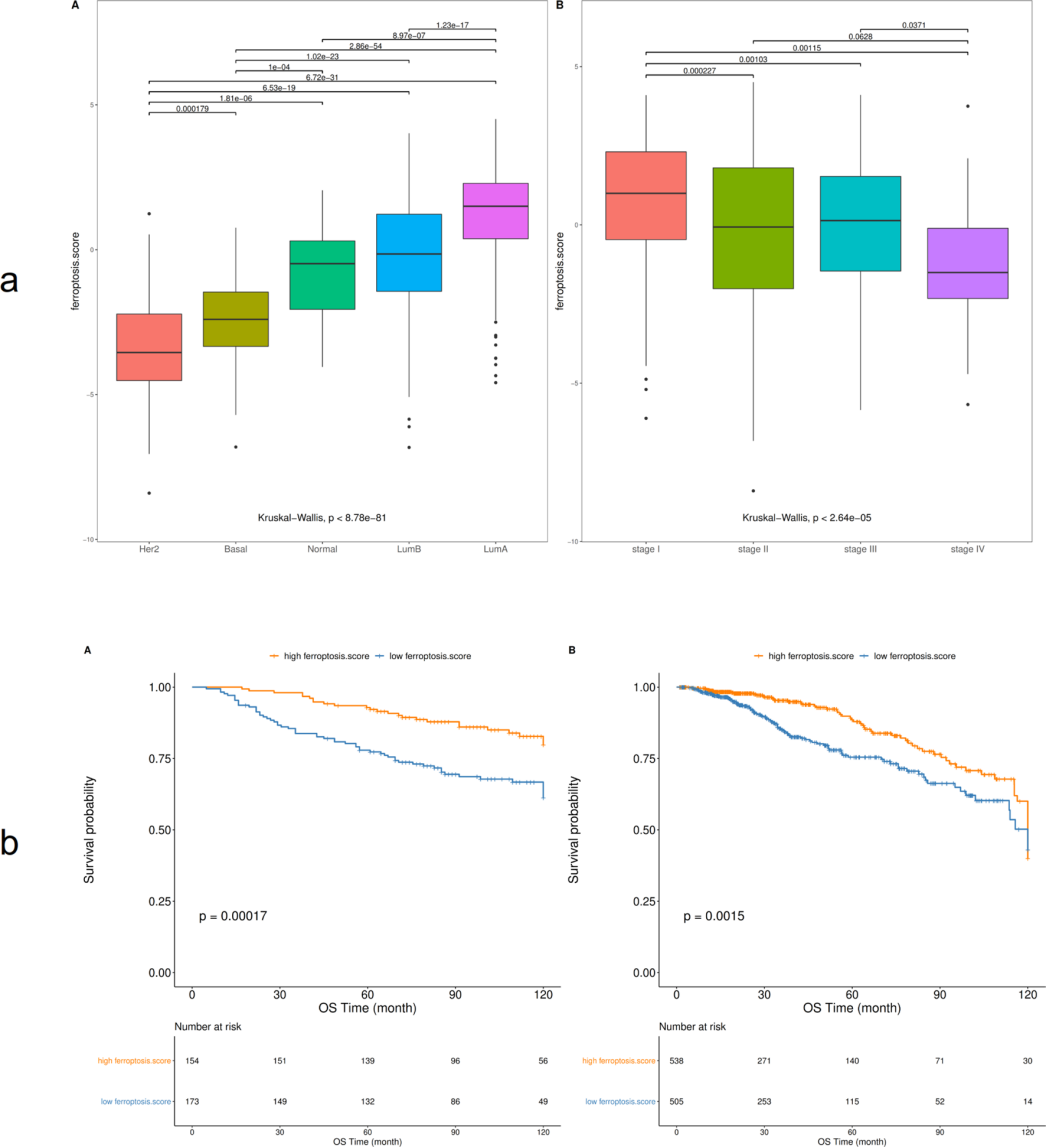
Relationship between ferroptosis scores with other datasets and prognosis. (**a**) Comparative analysis of ferroptosis scores in different PAM50 molecular classifications and clinical characteristics (A: PAM molecular classification, B: clinical stage); (**b**) Kaplan–Meier survival curve (A: GSE20685, B: TCGA).

Similarly, in the TCGA-BRCA dataset, the Wilcoxon test was used to analyze the differences in ferroptosis scores between different molecular subtypes and clinical characteristics. The results showed that ferroptosis scores differed significantly based on patient age and TP53 and PIK3CA gene mutations (Fig. 9). Significant differences were also found in different PAM50 molecular types and clinical stages (Fig. 10a). The Kaplan–Meier survival curve showed that the survival rate of the high ferroptosis score group in the GEO and TCGA datasets was significantly higher than that of the low ferroptosis score group (Fig. 10b).

### Analysis of molecular characteristics of high and low ferroptosis score groups in the TCGA dataset

**Figure 11:**
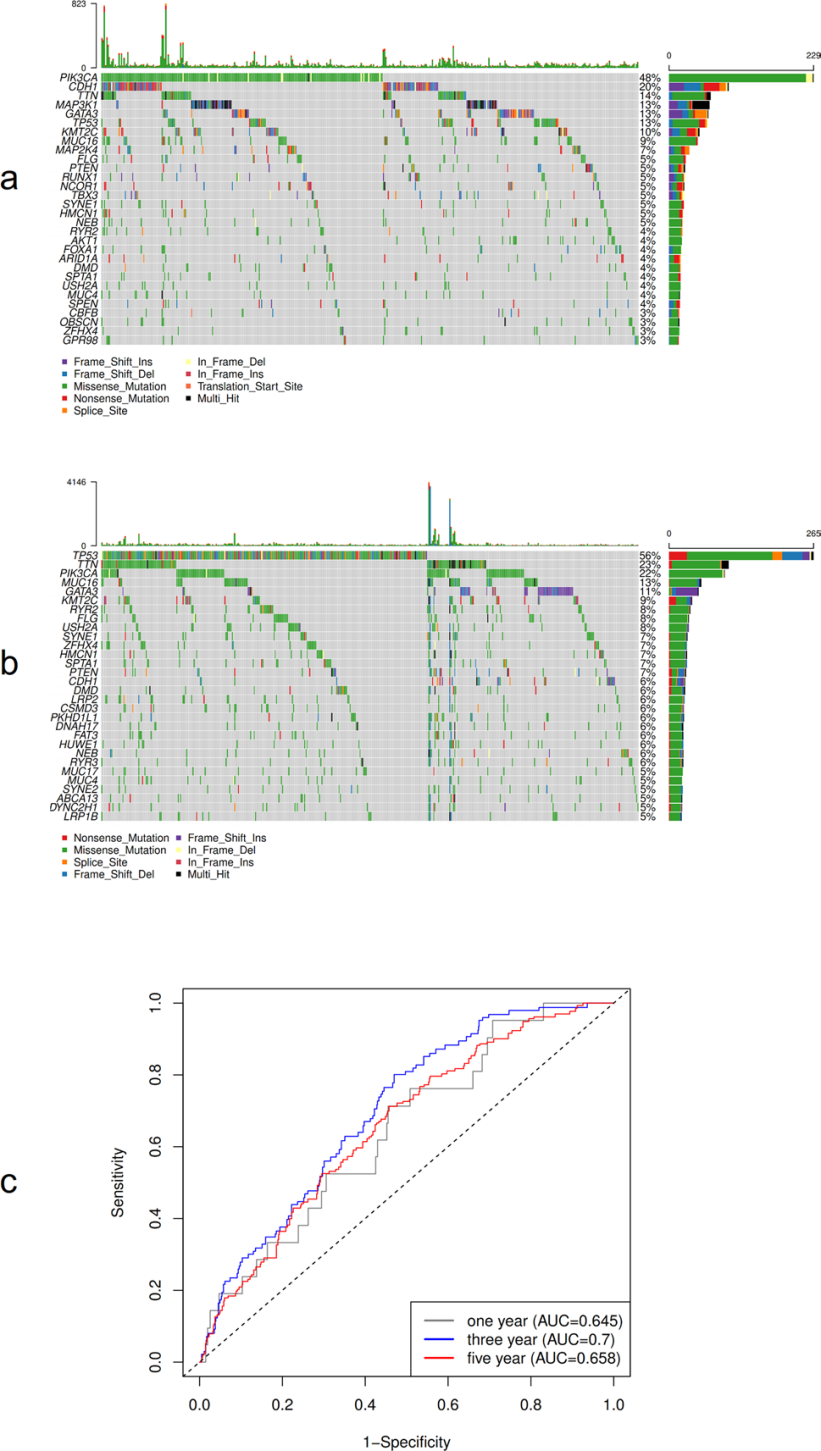
Molecular characteristics and prognosis of high and low ferroptosis scores. (**a**) The distribution of gene mutations in the high ferroptosis score group in the TCGA dataset; (**b**) The distribution of gene mutations in the low ferroptosis score group in the TCGA dataset; (**c**) Time-dependent ROC curve analysis.

In the TCGA dataset, we further explored the difference between the high and low ferroptosis score groups using the R package maftools to analyze the groups’ cell variation^28^. Figures 11a and b show the distribution of the top 30 with significant differences BRCA gene mutations in the two groups of samples.

### Predictive analysis of ferroptosis score on patients’ prognosis

The time-dependent receiver operating characteristic (ROC) curves of one-, three-, and five-year survival were analyzed based on the ferroptosis score model. The results showed that the area under the curve (AUC) of one, three, and five years were 0.645, 0.700, and 0.658, respectively. This shows that the ferroptosis model has high reliability in predicting the survival of patients (Fig. 11c).

## Discussion

The incidence of BRCA has risen to represent the highest incidence of female malignant tumors in the world and is increasing year by year. About 1.2 million women suffer from BRCA every year globally, which seriously endangers the physical and mental health of women^29,30^. Although the mortality rate of BRCA has been declining each year through early screening and diagnosis, the incidence rate is still the highest among female tumors^31^. BRCA has a good prognosis for solid tumors; the cure rate of early BRCA is 60–70%. Therefore, the prognostic indicators of BRCA are attracting increasing attention from researchers. Basic research in areas such as tumor genomics, RNA-omics, and proteomics is expanding application in clinical practice. Molecular pathological diagnosis is becoming increasingly important for early diagnosis, drug efficacy evaluation, and prognostic testing of BRCA, gradually changing the traditional empirical treatment model to an individual-body treatment model based on biomarkers^32–34^.

Ferroptosis is a highly iron-dependent mode of cell death that differs from apoptosis, necrosis, and other types of cell death in terms of genetic metabolism and molecular biology^35,36^. In recent years, ferroptosis has been a hotspot of tumor research, playing an important role in killing a variety of tumor cells. At present, most chemotherapeutic drugs act by inducing tumor cell apoptosis. If tumor cell apoptosis fails, however, chemotherapy resistance will occur. Overcoming chemotherapy resistance is urgent. As a new type of programmed cell death, ferroptosis has unique characteristics. The use of ferroptosis inducers and inhibitors has shown great potential in the treatment of tumors^37,38^. Activation of the ferroptosis pathway can induce cancer cell death^39^, especially in cases of drug resistance, and can enhance sensitivity to chemotherapeutics. For instance, cisplatin combined with ferroptosis inducer erastin can significantly improve anti-tumor activity, showing that ferroptosis can play an important role in tumor treatment^40^. Studies have shown that ferroptosis can be used as a new target for tumor suppression, opening up new methods for the treatment of clinical tumors^39,41^. A tumor treatment strategy based on ferroptosis is of great significance in inspiring the design of new drugs.

The cause of BRCA is not yet fully understood. Ferroptosis plays an important role in the death of BRCA cells, and the induction of ferroptosis in cancer cells has gradually become a treatment strategy for tumors. The relationship between ferroptosis-related genes and the prognosis of BRCA patients is not fully understood. To further study this relationship, this study systematically evaluated the mechanism of ferroptosis in BRCA, evaluated the predictive value of ferroptosis scores in BRCA on the prognosis of BRCA, and analyzed the clinicopathological characteristics and molecular types of BRCA patients. Through screening, 22 ferroptosis genes significantly related to the prognosis of BRCA were identified, and an interaction relationship between them was found. The unsupervised clustering results of 22 ferroptosis genes showed significant differences in prognosis between ferroptosis cluster A and ferroptosis cluster B. Ferroptosis cluster A was significantly enriched in biological pathways such as DNA replication and cell cycle. Ferroptosis cluster B was significantly enriched in biological pathways such as taurine metabolism. Almost all immune cell ratios and distributions between the two subgroups were different, and Natural.killer.cell, Activated.CD8.T.cell, Activated.B.cell, and Immature.B.cell were found to have a greater difference in cell prognosis. This suggests that ferroptosis is closely related to BRCA prognosis.

Several factors causing ferroptosis have been reported, such as sorafenib^42^, sulfasalazine^43^, artesunate^44^, and fluvastatin^45^, which are used to treat other diseases clinically, as well as small molecular substances such as erastin and rsl3^46^. Ma et al. found that a combination of siramesine and lapatinib induced ferroptosis by reducing the expression of ferroportin and increasing the expression of transferrin. Their findings may provide a new treatment method for anti-apoptotic BRCA cells^47^. This study further analyzed the potential biological behavior of each ferroptosis cluster. The results showed that the expression of most ferroptosis genes was significantly different among the groups of ferroptosis gene clusters, and Kaplan–Meier curves showed a significant relationship between ferroptosis gene cluster and OS rate and that the prognosis of Type A tumors in ferroptosis gene clusters was poor. Subsequently, single-factor Cox regression analysis and random forest dimensionality reduction were used to screen for characteristic genes to construct a ferroptosis score model. Finally, 9 ferroptosis-related genes with prognostic value were found, genetic prognostic models were constructed (BCL2, SUSD3, SERPINA3, AGBL2, SEC14L2, ELOVL2, FGD3, CASC1, TPRG1), and the relationship between ferroptosis score and BRCA prognosis was analyzed. The results showed that the OS rate of the high ferroptosis score group was significantly higher than that of the low ferroptosis score group. The results of time-dependent ROC curve analysis of patients’ one-, three-, and five-year survival rates based on the ferroptosis score model showed that the model had high reliability in predicting patients’ survival. This shows that calculations based on ferroptosis scores can characterize the prognosis of a sample well.

In addition, the clinicopathological characteristics and molecular classification of the ferroptosis score model were analyzed, and the results showed significant differences regarding clinical stage and patient age in ferroptosis cluster subgroups. Low ferroptosis score was concentrated mainly in more advanced tumor stages, and patients older than 65 years and with less advanced tumor stages were more concentrated in the high ferroptosis score group, which further confirmed that the high ferroptosis score group had a better prognosis.

The correlation analysis between ferroptosis score and known gene characteristics showed that ferroptosis score was significantly positively correlated with biological functions such as DNA replication, cell cycle, and DNA mismatch repair. Mutations of TP53 and PIK3CA genes were among the most common mutations in BRCA, but the study about the role of TP53 and PIK3CA mutations in treatment response and survival of BRCA was still uncertain. Ungerleider et al.^48^ used a large data set to examine the overall survival rate according to the TP53 mutation status of patients across multiple clinical features and treatments, and found that the 5-year survival probability of patients with TP53-mutated tumors was significantly reduced. Silwal-Pandit et al.^49^ proposed that somatic mutation of the TP53 gene is a strong prognostic marker for BRCA, and their study found that TP53 mutations were associated with increased mortality in patients with luminal B, HER2-enriched, and normal-like tumors, and showed that TP53 mutations have different clinical relevance in molecular subtypes of BRCA. This study analyzed the differences in ferroptosis score in molecular subtypes and clinical characteristics of TP53 and PIK3CA. The results showed that ferroptosis score differed significantly in TP53 and PIK3CA gene mutations. TP53 mutated type (TP53.Mut) and PIK3CA wild type (PIK3CA.WT) showed lower ferroptosis scores, Kaplan–Meier curve analysis showed that the overall survival rate of the ferroptosis score low group was lower, indicating that patients with TP53.Mut and PIK3CA.WT may tend to have a lower survival rate,showing that the ferroptosis score seems to be a protective factor. The study by Maruyama et al.^50^ reported that PIK3CA mutation was associated with prognosis, and PIK3CA mutant had better prognosis, which was similar to the results of this study. The analysis of ferroptosis scores in different PAM50 molecular types and clinical stages found that the ferroptosis scores of Her2 and Basal molecular types were lower than LumB and LumA types, while stage IV tumors and Her2 molecular types showed the lowest ferroptosis scores, suggesting that patients with Her2 and Basal molecular types and stage IV tumors may have a poor prognosis. The report by Raza et al.^51^ also proposed that HER2 is the malignant tumor with the worst prognosis, indicating that ferroptosis score may have a predictive advantage for precise immunotherapy of BRCA. In addition, Kaplan–Meier survival curves showed that the survival rate of the high ferroptosis score group in the GEO and TCGA datasets was significantly higher than that of the low ferroptosis score group, indicating that ferroptosis score may be a valuable prognostic factor for BRCA patients. Ferroptosis score can thus be used to comprehensively assess the clinicopathological features of patients, including tumor differentiation, clinical stage, cell infiltration, and molecular subtypes. Similarly, ferroptosis score can be used as an independent prognostic biomarker for the survival of BRCA patients, providing some new insights and ideas for the diagnosis and treatment of BRCA.

There are some limitations in this study. Firstly, Although the total number of samples in this study reached 1468, the number of validated cases was not sufficient. Secondly, It is worth noting that the data of this study come from the TCGA-BRCA database, and its accuracy and credibility need to be further confirmed. On the other hand, our method is mainly based on gene expression data analysis and fails to take into account other factors (methylation, copy number variation, proteomic data, etc.).

## Conclusion

Ferroptosis activation plays a non-negligible role in tumor occurrence and development. This study provides valuable prognostic factors and prognostic models for BRCA patients, and evaluating the ferroptosis score within BRCA will help advance our understanding of the infiltrating properties of cells in the tumor microenvironment and may guide more effective immunotherapy strategies.

## Supplementary Information

Supplementary Information 1.

Supplementary Information 2.

Supplementary Information 3.

Supplementary Information 4.

Supplementary Information 5.

Supplementary Information 6.

Supplementary Information 7.

## Data Availability

The RNA sequencing data and clinicopathological and survival data of TCGA-BRCA were downloaded from UCSC Xena database (https://xena.ucsc.edu/). In the GEO database (https://www.ncbi.nlm.nih.gov/geo/) number GSE20685, the expression profile of the BRCA population and clinical data were selected, and the GEO. The expression profile and clinical data of French BRCA patients were obtained from the ICGC database (https://dcc.icgc.org/releases/current/Projects/BRCA-FR). The c2.cp.kegg.v7.1 gene set was downloaded from the MsigDB database (https://www.gsea-msigdb.org/gsea/msigdb/index.jsp). All the datasets were open access datasets.
