# Supplementary material for: Predicting prognosis and clinical features of the tumor microenvironment based on ferroptosis score in patients with breast cancer
Source: Sci Rep. 2022 Jun 23;12:10611. doi: 10.1038/s41598-022-14964-7 (PMC9226039; doi:10.1038/s41598-022-14964-7)
Supplement: Supplementary file 7 — Supplementary Information 7. [file 41598_2022_14964_MOESM7_ESM.docx]

**The Supplementary Table Legends**

Supplementary Table 1. 15,343 gene expression profiles.

Supplementary Table 2. 176 Ferroptosis related genes.

Supplementary Table 3. 516 differential genes.

Supplementary Table 4. 9 ferroptosis phenotype–related genes.

Supplementary Table 5. The information of KEGG_GSVA_wilcox.

Supplementary Table 6. The information of KEGG_GSVA.
